# Physicians are a key to encouraging cessation of smoking among people living with HIV/AIDS: a cross-sectional study in the Kathmandu Valley, Nepal

**DOI:** 10.1186/1471-2458-11-677

**Published:** 2011-08-31

**Authors:** Rachel M Amiya, Krishna C Poudel, Kalpana Poudel-Tandukar, Jun Kobayashi, Basu D Pandey, Masamine Jimba

**Affiliations:** 1 Department of Community and Global Health, Graduate School of Medicine, University of Tokyo, Tokyo, Japan; 2 Waseda Institute for Advanced Study, Waseda University, Tokyo, Japan; 3 Bureau of International Cooperation, National Center for Global Health and Medicine, Tokyo, Japan; 4 Sukraraj Tropical and Infectious Disease Hospital, Kathmandu, Nepal

## Abstract

**Background:**

HIV care providers may be optimally positioned to promote smoking behaviour change in their patients, among whom smoking is both highly prevalent and uniquely harmful. Yet research on this front is scant, particularly in the developing country context. Hence, this study describes smoking behaviour among people living with HIV/AIDS (PLWHA) in the Kathmandu Valley of Nepal, and assesses the association between experience of physician-delivered smoking status assessment and readiness to quit among HIV-positive smokers.

**Methods:**

We conducted a cross-sectional survey of PLWHA residing in the Kathmandu Valley, Nepal. Data from 321 adult PLWHA were analyzed using multiple logistic regression for correlates of current smoking and, among current smokers, of motivational readiness to quit based on the transtheoretical model (TTM) of behaviour change.

**Results:**

Overall, 47% of participants were current smokers, with significantly higher rates among men (72%), ever- injecting drug users (IDUs), recent (30-day) alcohol consumers, those without any formal education, and those with higher HIV symptom burdens. Of 151 current smokers, 34% were thinking seriously of quitting within the next 6 months (contemplation or preparation stage of behaviour change). Adjusting for potential confounders, experience of physician-delivered smoking status assessment during any visit to a hospital or clinic in the past 12 months was associated with greater readiness to quit smoking (AOR = 3.34; 95% CI = 1.05,10.61).

**Conclusions:**

Roughly one-third of HIV-positive smokers residing in the Kathmandu Valley, Nepal, are at the contemplation or preparation stage of smoking behaviour change, with rates significantly higher among those whose physicians have asked about their smoking status during any clinical interaction over the past year. Systematic screening for smoking by physicians during routine HIV care may help to reduce the heavy burden of smoking and smoking-related morbidity and mortality within HIV-positive populations in Nepal and similar settings.

## Background

As expanding global application of antiretroviral therapy (ART) heralds a paradigm shift in HIV-related morbidity and mortality [1-5], the time is ripe to address tobacco smoking as a bottleneck to gains in life expectancy among people living with HIV/AIDS (PLWHA) [6,7]. Recent studies from HIV-positive cohorts in developed country settings indicate that rates of current smoking (41-66%) [7-10] are two to three times higher than corresponding general population rates, and similarly elevated smoking rates have been observed among PLWHA in developing African countries [11-14]. In addition to accelerating the development of well-known tobacco-associated morbidities [15], smoking weakens the body's immune and virological response [16], rendering HIV-positive smokers more vulnerable to disease and opportunistic infection and less responsive to ART [17,18]. PLWHA who smoke report lower quality of life and face significantly elevated risks of non-AIDS cancers, cardiovascular disease, bacterial pneumonia, and all-cause mortality relative to non-smoking PLWHA [8,10,19-21].

Yet even for individuals with protracted tobacco use histories, cessation brings almost immediate health benefits and can, over time, reduce the risk of premature death to a level approaching that of a lifetime non-smoker [22,23]. From an HIV-specific perspective, evidence confirms that quitting smoking can dramatically relieve symptom burden [24] and improve health outcomes, including reduced risk of bacterial pneumonia [25] and cardiovascular disease [26]. Moreover, facilitation of smoking cessation alongside HIV treatment may help prevent medication failures and substance use relapse [27,28].

A key component and predictor of successful outcomes in the process of smoking cessation is motivational readiness to change [29,30], described in the transtheoretical model (TTM) of health behaviour change [31,32]. In the case of smoking, one study suggests that advancement from precontemplation (not thinking of quitting within the next 6 months) to contemplation (thinking of quitting in the next 1-6 months) increases the likelihood of future (2-year) cessation by 40%, and that advancement to preparation (thinking of quitting within the next 30 days, with at least one 24-hour quit attempt in the past year) increases that likelihood by 80% [30]. Among HIV-positive smokers, several cross-sectional studies carried out in the United States report that 58-64% of those treated in outpatient clinics are thinking seriously of quitting within the next 6 months [9,33-35], indicating a certain readiness and likelihood to proceed through the remaining stages of change. Understanding what characteristics or inputs might impact readiness to quit among HIV-positive smokers is key to devising effective strategies to promote cessation.

One promising vehicle for reaching HIV-positive smokers may lie in the frequent contact between HIV-positive smokers and their physicians facilitated by the need for regular immunologic monitoring and, for many, ART support. Given this heightened level of interaction and trust [36], HIV medical systems and personnel would seem ideally placed to effect behaviour change in this important population of smokers. Indeed, a previous study carried out among state employees in the United States identified patients' trust in their physician and physicians' knowledge of patients as leading correlates of adherence to physician's advice [37].

Within the general population, even simple interventions delivered by physicians in the form of brief encouragement or advice to quit smoking can have a significant impact on short-term movement through the stages of change [38] and on future quitting rates [39]. However, the effect of such basic interventions remains unclear in the context of HIV care systems, where the space for positive impact may be even greater. If shown effective, the implementation of such basic awareness-raising measures could represent an important first step toward tackling the troubling intersection of HIV and tobacco epidemics, particularly in the hard-hit and resource-limited countries of the developing world.

Despite the exceptional health risks associated with tobacco smoking and the clear benefits of quitting for HIV-positive smokers, attention to smoking behaviour and motivational readiness to quit has been inadequate in this important smoking subgroup. Moreover, although over 90% of the world's HIV-positive population resides in the developing world [40], data on tobacco use among PLWHA outside of Europe and North America have been limited to several descriptive reports from Africa [11-14].

We therefore explored factors associated with current smoking status and readiness to quit among PLWHA residing in the Kathmandu Valley of Nepal, among the least developed countries in South Asia. In particular, this study examined the association between experience of physician-delivered smoking status assessment and motivational readiness to quit in HIV-positive smokers.

## Methods

### Study design and setting

This cross-sectional study surveyed a community-based sample of 321 HIV-positive residents of the Kathmandu Valley, Nepal. Since the country's first case of HIV was reported in 1988, Nepal has been facing a burgeoning concentrated epidemic, with prevalence especially high among injecting drug users (IDUs) [41]. In the capital city of Kathmandu, HIV prevalence among IDUs was 34.8% in 2007 [42]. The Kathmandu Valley comprises three districts (Kathmandu, Lalitpur, and Bhaktapur) with an estimated population of 2.2 million [43]. Of Nepal's estimated 70,000 adult (15-49 years) PLWHA [41], 15.7% were residing in the Kathmandu Valley at the end of 2006 [44].

### Data collection

We recruited 322 participants through purposive, convenience sampling techniques. Potential participants were referred through staff members of five local non-governmental organizations (NGOs) working within HIV-positive communities in the Kathmandu Valley. Individuals recruited for participation fulfilled the following inclusion criteria: (1) 16 to 60 years of age, (2) self-reported diagnosis of HIV-positive status, and (3) willing provision of written informed consent for voluntary participation.

Data collection was undertaken during February and March 2010 in the wider context of the baseline phase of a longitudinal healthy living intervention overseen by the second author (KCP). The survey was based on a structured, pre-tested Nepali language questionnaire. Trained interviewers administered the questionnaire face-to-face in a private setting, with each interview lasting approximately 45-60 minutes. All participants were informed about the study procedures using a prepared information sheet, and signed informed consent forms prior to being interviewed. To enhance confidentiality, interviewers reassured participants that numerical codes would be used in place of names in all records. The study protocol was reviewed and ethical approval granted by the Institutional Review Board of the Graduate School of Medicine at the University of Tokyo and by the Nepal Health Research Council.

### Measures

#### Sociodemographic, clinical, and psychosocial characteristics

Standard single questionnaire items assessed basic sociodemographic and HIV-specific clinical variables. We used the HIV Symptom Index (HSI) [45], which assesses the presence and degree of 20 symptoms commonly experienced by PLWHA, to measure HIV symptom burden (Cronbach's alpha = 0.89). Participants reported whether each symptom was present, and if so, whether it was bothersome, by using a five-point Likert scale (0-4) ranging from "I do not have this symptom" (0) to "I have it and it bothers me a lot" (4). We dichotomized each item into absent/not bothersome (0-2) vs. present and bothersome (3-4), and summed these scores to obtain a bothersome symptom count [45].

A modified 7-item version of the AIDS-related Stigma Scale [46] was used to assess internalized AIDS stigma (Cronbach's alpha = 0.74). Responses were given dichotomously (0 = disagree, 1 = agree); scale scores represent the sum total of endorsed items, with higher scores indicating more negative attitudes or perceived discrimination. Additionally, the 21-item Beck Depression Inventory (BDI), Nepali version [47,48], was used to assess depression in participants (Cronbach's alpha = 0.90).

#### Smoking-related variables

Current smoking status was assessed by single items asking participants about smoking frequency at the time of the survey and about amount smoked both daily and cumulatively. Based on definitions set forth by the CDC [49], we classified participants as *current smokers *if they reported smoking "every day" or "some days" at the time of survey. Participants who had smoked at least 100 cigarettes in their lifetime but had quit smoking at the time of the interview were considered *former smokers*, while those reporting never smoking or smoking fewer than 100 cigarettes in their lifetime were considered *never smokers*.

Frequency of smoking as a measure of smoking intensity was assessed by asking current smokers how many cigarettes they smoked in a typical day. Current smokers were then categorized as *light smokers *(≤ 10 cigarettes/day) or *moderate-to-heavy smokers *(> 10 cigarettes/day) [50]. Current smokers were also asked whether they smoked fewer, more, or about the same number of cigarettes since finding out they were HIV-positive [51]. We identified previous quit attempts by asking current smokers whether they had stopped smoking for at least one day during the past 12 months because they were trying to quit smoking. Additional items addressed to current smokers covered age of smoking initiation and cohabitation with other smokers.

#### Health care provider-delivered smoking status assessment

Experience of smoking status assessment by a health care provider was measured by first asking all participants their frequency of visits to a hospital or clinic for any reason over the 12 months preceding the time of survey. Those reporting at least one visit to a hospital or clinic during that time (n = 302) then responded to three items inquiring whether a physician, paramedic, or nurse had ever asked about their smoking status during any such visit.

#### Readiness to quit smoking

Current smokers' readiness to quit smoking was assessed based on the following question: "Are you seriously thinking of quitting smoking?" According to the conventions of the TTM [29], participants reporting no intention to quit within the next 6 months or no intention to quit at all were placed in the *pre-contemplation *stage, while those reporting an intention to quit in the next 1-6 months were classified as falling within the *contemplation *stage. Those expressing an intention to quit within the next 30 days and also reporting a previous quit attempt in the past 12 months were categorized in the *preparation *stage of change.

### Statistical analysis

Of 322 eligible participants, data from one male participant was incomplete and thus excluded from analysis. The remaining 321 HIV-positive individuals (184 males, 137 females) constituted our study sample.

Multiple logistic regression analysis was used to explore factors associated with smoking status and to examine the association between experience of physician-delivered smoking status assessment and readiness to quit smoking, comparing current smokers in lower (pre-contemplators) and higher (contemplators and preparators) stages of change in terms of potential covariates. Independent variables were entered into each regression using a direct (simultaneous) entry method. Major sociodemographic characteristics and other mediators having previously established or theoretically feasible associations with the dependent variables were included as covariates or potential confounders in the analyses. Multicollinearity was also assessed for each model, and the variance inflation factor (VIF) of all variables was less than 3.0. All statistical tests were 2-sided, considered significant at the p < 0.05 level, and performed using SPSS version 18.0 for Windows (SPSS Inc., Chicago, IL).

## Results

### Background characteristics

The surveyed group was 57% male and had a median age of 33 (interquartile range [IQR] = 30, 39) years; 81% of participants had at least some formal education (Table 1). Median period since testing HIV-positive was 53 (IQR = 25, 84) months and 73% of participants were on ART at the time of survey. Median bothersome HIV symptom count was 3 (IQR = 1, 7), and around a quarter (26%) of participants registered moderate-to-severe depression. Overall, 41% of participants had a lifetime history of injecting drug use.

**Table 1:** Background characteristics of participants (N = 321)

Characteristic	**n**^a^	(%)
**Sociodemographics**		
Gender		
Male	137	(57.3)
Female	184	(42.7)
		
Age (years)		
20-29	78	(34.2)
30-39	165	(47.0)
40-60	76	(18.8)
		
Current marital status		
Unmarried	101	(31.5)
Married	220	(68.5)
		
Education level^b^		
No formal education	58	(18.6)
Primary (1-5 yrs.)	68	(21.9)
Lower secondary (6-10 yrs.)	156	(50.2)
Higher secondary and above (11+ yrs.)	29	(9.3)
		
Monthly income (NRs)^c ^(Median = 4000)		
0	89	(28.3)
200-4000	80	(25.4)
4001-300000	146	(46.3)
		
Any children		
No	102	(31.8)
Yes	219	(68.2)
		
Sexual orientation		
Heterosexual	319	(99.4)
Homo- or bi-sexual	2	(0.6)
		
**Clinical and psychosocial characteristics**		
Months since first testing HIV+ (Median = 53)		
1-53	158	(50.0)
54+	158	(50.0)
		
Months on ART (Median = 24)		
Not currently on ART	87	(27.2)
1-24	129	(40.3)
25+	104	(32.5)
		
Experience of TB diagnosis since testing HIV+		
No	236	(73.5)
Yes	85	(26.5)
		
Bothersome HIV symptom count (Median = 3)		
0-3	173	(53.9)
4-20	148	(46.1)
		
Smoking status		
Never smoker	129	(40.2)
Former smoker	40	(12.5)
Current smoker	152	(47.4)
		
Injection drug use history^d^		
Never user	191	(60.1)
Former user	100	(31.4)
Current user	27	(8.5)
		
Alcohol use, last 30 days		
No	281	(87.5)
Yes	40	(12.5)
		
Marijuana use, last 6 months		
No	290	(90.3)
Yes	31	(9.7)
		
Internalized AIDS stigma score (Median = 4)		
0-4	187	(58.3)
5-7	134	(41.7)
		
Beck Depression Inventory score^e^		
< 20	239	(74.5)
20+	82	(25.5)

*NRs*, Nepalese Rupees. *ART*, antiretroviral therapy.

^a ^Total sample size varies due to missing data.

^b ^Based on the structure of the Nepalese education system.

^c ^4000 NRs = US $55 approximately, as of February 2010.

^d ^*Former users*: participants reporting a lifetime history of injecting drug use but no use in the last 6 months classified. *Current users*: participants reporting injecting drug use in the last 6 months. *Never users*: participants reporting no lifetime history of injecting drug use. Three participants who indicated a lifetime history of injecting drug use did not respond to the question regarding 6-month use and hence could not be distinguished as former or current users.

^e ^A score of 20 or more on the Beck Depression Inventory defined the presence of moderate-to-severe depressive symptoms, based on clinical validation of the scale in Nepal (sensitivity = 0.73, specificity = 0.91) [48].

### Smoking prevalence and characteristics

Of the 321 participants, 47% were current smokers at the time of survey - 72% of male and 15% of female participants. Median age of smoking initiation among the 152 current smokers was 14 years (IQR = 12, 17 years), and roughly half (51%) were moderate-to-heavy smokers (Table 2). Nearly two-thirds (62%) of all current smokers reported some change in their level of smoking since testing HIV-positive, with 45% reporting a reduction and 17% reporting an increase. A total of 23 participants (19 men, 4 women) had quit smoking subsequent to testing HIV-positive.

**Table 2:** Tobacco use characteristics of current smokers (N = 152)

Characteristic	**n**^a^	(%)
Age of smoking initiation (years) (Median = 14)		
7-14	78	(51.7)
15+	73	(48.3)
		
Average daily smoking intensity, last 30 days		
Light smoker (≤ 10 cigarettes/day)	75	(49.3)
Moderate-to-heavy smoker (> 10 cigarettes/day)	77	(50.7)
		
Presence of other smoker in household		
No	70	(46.1)
Yes	82	(53.9)
		
Change in smoking level since testing HIV+		
Cut down	68	(44.7)
About the same	58	(38.2)
Increased	26	(17.1)
		
Readiness to quit smoking (Stage of change)		
Pre-contemplation	100	(66.2)
Contemplation	26	(17.2)
Preparation	25	(16.6)
		
Prior quit attempt, last 12 months		
No	74	(48.7)
Yes	78	(51.3)

^a ^Total numbers of respondents vary due to missing data.

Regarding readiness to quit, 51 current smokers (34%) had reached or surpassed the contemplation stage of change, with some thought of quitting within the next six months. Roughly half (52%) of current smokers had made at least one quit attempt within the past 12 months; the most commonly cited reason for relapse was "Addiction/Habit" (45%), followed by "Boredom" (32%).

Among the 137 current smokers reporting at least one visit to a hospital or clinic within the past 12 months, 73% reported experience of physician-delivered smoking status assessment during any such visit. Further, 26% reported experience of paramedic- or nurse-delivered smoking status assessment within the same parameters.

### Factors associated with current smoking

Men were over nine times more likely than women to be current smokers (AOR = 9.20; 95% CI = 3.80, 22.26) (Table 3). Additionally, current smoking was nearly six times as prevalent among participants with a lifetime history of injecting drug use (AOR = 5.72; 95% CI = 2.74, 11.92), over three times as prevalent among those reporting any alcohol consumption in the past 30 days (AOR = 3.19; 95% CI = 1.10, 9.30), and twice as prevalent among those with higher HIV symptom burdens (AOR = 2.06; 95% CI = 1.04, 4.10). Conversely, participants having any level of formal education were nearly three times *less *likely than those without any formal education to be current smokers (AOR = 0.35; 95% CI = 0.14, 0.84).

**Table 3:** Multiple logistic regression analysis of factors associated with current smoking^a ^among participants (N = 301^b^)

Variable	AOR	(95% CI)
Gender		
Male	9.20	(3.80, 22.26)**
Female (Ref)	1.00	
		
Age (years)		
34+	0.72	(0.36, 1.43)
20-33 (Ref)	1.00	
		
Marital status		
Married	0.92	(0.43, 1.96)
Unmarried (Ref)	1.00	
		
Any formal education		
Yes	0.35	(0.14, 0.84)*
No (Ref)	1.00	
		
Monthly income (NRs)^c^		
4001+	0.74	(0.39, 1.40)
0-4000 (Ref)	1.00	
		
Any children		
Yes	1.03	(0.49, 2.16)
No (Ref)	1.00	
		
Months since first testing HIV+		
54+	1.51	(0.79, 2.89)
1-53 (Ref)	1.00	
		
Currently receiving ART		
Yes	0.77	(0.36, 1.65)
No (Ref)	1.00	
		
Bothersome HIV symptom count		
4-20	2.06	(1.04, 4.10)*
0-3 (Ref)	1.00	
		
Experience of TB diagnosis since testing HIV+		
Yes	0.73	(0.40, 1.48)
No (Ref)	1.00	
		
Internalized AIDS stigma score		
5-7	0.84	(0.44, 1.62)
0-4 (Ref)	1.00	
		
Beck Depression Inventory score^d^		
20+	0.49	(0.22, 1.11)
< 20 (Ref)	1.00	
		
Lifetime injecting drug use		
Yes	5.72	(2.74, 11.92)**
No (Ref)	1.00	
		
Alcohol consumption, last 30 days		
Yes	3.19	(1.10, 9.30)*
No (Ref)	1.00	
		
Marijuana use, last 6 months		
Yes	2.59	(0.73, 9.15)
No (Ref)	1.00	
		
Presence of smoker among other household members		
Yes	1.09	(0.58, 2.02)
No (Ref)	1.00	

*AOR*, adjusted odds ratio. *CI*, confidence interval. *ART*, antiretroviral therapy.

^a ^The reference group was current non-smokers.

^b ^After accounting for the collective set of missing values associated with the variables included in the multivariable model, data from 301 participants were analyzed.

^c ^4000 NRs = US $55 approximately, as of February 2010.

^d ^A score of 20 or more on the Beck Depression Inventory defined the presence of moderate-to-severe depressive symptoms, based on clinical validation of the scale in Nepal (sensitivity = 0.73, specificity = 0.91) [48].

*p < 0.05; **p < 0.01.

### Factors associated with readiness to quit smoking

Experience of physician-delivered smoking status assessment during any visit to a hospital or clinic in the last 12 months was significantly associated with higher readiness to quit among the surveyed HIV-positive smokers (AOR = 3.34; 95% CI = 1.05, 10.61) (Table 4). Additionally, current smokers reporting at least one prior quit attempt in the past 12 months were significantly more likely than those reporting no such attempt to be contemplating quitting within the next 6 months (AOR = 3.77; 95% CI = 1.48, 9.58), as were smokers having at least one other regular smoker in the household (AOR = 2.91; 95% CI = 1.16, 7.29). Conversely, having any formal education was significantly associated with being in the precontemplative stage of behaviour change, showing an inverse relationship with readiness to quit (AOR = 0.12; 95% CI = 0.02, 0.71).

**Table 4:** Multiple logistic regression analysis of factors associated with readiness to quit (contemplation or preparation stage of change)^a ^among current smokers visiting a hospital or clinic in the past 12 months (N = 131^b^)

Variable	AOR	(95% CI)
Gender		
Male	5.79	(0.86-39.10)
Female (Ref)	1.00	
		
Age (years)		
34+	1.10	(0.40-3.03)
20-33 (Ref)	1.00	
		
Marital status		
Married	1.30	(0.39-4.30)
Unmarried (Ref)	1.00	
		
Any formal education		
Yes	0.12	(0.02-0.71)*
No (Ref)	1.00	
		
Monthly income (NRs)^c^		
4001+	0.97	(0.39-2.41)
0-4000 (Ref)	1.00	
		
Any children		
Yes	1.30	(0.42-4.05)
No (Ref)	1.00	
		
Months since first testing HIV+		
54+	0.97	(0.37-2.49)
1-53 (Ref)	1.00	
		
Currently receiving ART		
Yes	0.71	(0.26-1.93)
No (Ref)	1.00	
		
Bothersome HIV symptom count		
4-20	0.50	(0.19-1.32)
0-3 (Ref)	1.00	
		
Experience of TB diagnosis since testing HIV+		
Yes	0.80	(0.25-2.51)
No (Ref)	1.00	
		
Number of hospital visits, last 12 months		
3+	0.87	(0.32-2.39)
1-2 (Ref)	1.00	
		
Beck Depression Inventory score^d^		
20+	1.20	(0.41-3.57)
< 20 (Ref)	1.00	
		
Lifetime injecting drug use		
Yes	0.50	(0.16-1.60)
No (Ref)	1.00	
		
Presence of smoker among other household members		
Yes	2.91	(1.16-7.29)*
No (Ref)	1.00	
		
Age of smoking initiation (years)		
15+	1.67	(0.67-4.15)
7-14 (Ref)	1.00	
		
Average daily smoking intensity, last 30 days		
Moderate-to-heavy smoker (> 10 cigarettes/day)	0.42	(0.16-1.13)
Light smoker (≤ 10 cigarettes/day) (Ref)	1.00	
		
Prior quit attempt, last 12 months		
Yes	3.77	(1.48-9.58)**
No (Ref)	1.00	
		
Experience of smoking status assessment by a physician, last 12 months		
Yes	3.34	(1.05-10.61)*
No (Ref)	1.00	
		
Experience of smoking status assessment by paramedic or nurse, last 12 months		
Yes	1.01	(0.36-2.85)
No (Ref)	1.00	

*AOR*, adjusted odds ratio. *CI*, confidence interval. *ART*, antiretroviral therapy.

^a ^The reference group was current smokers in the precontemplation stage of change.

^b ^After accounting for the collective set of missing values associated with the variables included in the multivariable model, data from 131 participants were analyzed.

^c ^4000 NRs = US $55 approximately, as of February 2010.

^d ^A score of 20 or more on the Beck Depression Inventory defined the presence of moderate-to-severe depressive symptoms, according to clinical validation of the scale in Nepal (sensitivity = 0.73, specificity = 0.91) [48].

*p < 0.05; **p < 0.01.

## Discussion

This study revealed that HIV-positive smokers reporting experience of physician-delivered smoking status assessment during any visit to a hospital or clinic in the past 12 months were over three times more likely to be in the contemplation or preparation stage of readiness to quit smoking. Outside of gender, lifetime history of injecting drug use showed the most robust association with current smoking status, followed by 30-day alcohol consumption, lack of formal education, and HIV symptom burden. Thus, optimal targeting of high-risk groups for smoking cessation efforts within the HIV-positive community would focus especially on males and recovering or active IDUs, incorporating the physician as a viable conduit for smoking cessation interventions.

As expected, current smoking was highly prevalent among PLWHA in our study, particularly among male participants. Though our data reveal a current smoking prevalence among HIV-positive women roughly comparable to the national rate for women in the region (16.0%), the prevalence of smoking among HIV-positive men was over twice the corresponding national rate (34.8%) [52]. Identified predictors of current smoking status were generally consistent with results of previous studies conducted within HIV-positive populations in Western, developed settings [10,33] and in Africa [11].

In terms of motivational readiness to quit, the proportion of participants still in the precontemplative stage of behaviour change was high relative to previous findings on HIV-positive smokers in developed countries [9,33,34]. Velicer et al. [53] reported typical proportions for readiness to quit as 40% in precontemplation, 40% in contemplation, and 20% in preparation among smokers in the general population. In our sample, in contrast, roughly two-thirds of smokers were precontemplative about quitting. This finding underscores the prime importance of exploring effective ways to build motivation for cessation in this important population of smokers.

Our study is the first to report on formal education as a potential barrier to quitting readiness among HIV-positive smokers. Given the strong positive linear correlation between education and the quit ratio (proportion of ever smokers who had quit smoking) demonstrated by Wetter et al. [54], it may be that those formally educated individuals still smoking at the time of our survey were less likely to be thinking seriously of quitting because they had already made a conscious decision to continue smoking, fully cognizant of the attendant risks. Further research would be warranted to elucidate the observed association between education level and readiness to quit smoking among PLWHA and in the developing country context.

Similarly, though data from developed countries suggest that smokers attempting to quit may be less successful and more susceptible to relapse with the presence of another smoker in the household [55,56], our data indicate that cohabitation with at least one other smoker may contribute to greater quitting readiness in some populations. Indeed, social support networks comprise an important aspect of successful smoking cessation [57]. Although this study did not specifically address the readiness to quit of smoking family members, we can reasonably assume that some discussion of quitting is likely to take place where more than one smoker inhabits a household. Future research might fruitfully explore cessation interventions designed to exploit existing support systems through soliciting the involvement of other household smokers.

We further found that history of at least one smoking cessation attempt was associated with greater quitting readiness. This finding is consistent with the TTM, by which the probability of successful change increases with the number of change attempts [32], and is also supported by results from previous studies of HIV-positive smokers [9]. For the promotion of active progression through the stages of change toward cessation, this result recommends the importance of encouraging quit attempts even among those smokers demonstrating lower levels of motivational readiness.

Finally, our research opens up a new and previously unexplored space for the role and effectiveness of basic smoking status screening administered by physicians treating PLWHA. Because the HIV practice pattern typically consists of regular contact over an extended period of time, physicians treating PLWHA have enhanced opportunities to build rapport and trust and to better tailor the tone to the individual patient. Yet there is evidence that HIV care providers may actually be *less *likely to recognize current smoking in their patients [51,58]. This points to a critical missed opportunity.

Notably, we found significantly higher motivational readiness for cessation among individuals reporting smoking status assessment by a physician, though not among individuals reporting such assessment by a paramedic or nurse. This implies that, in the context of this study setting, the words of a physician may be imbued with greater meaning or weight when it comes to issues of health behaviour change. Specifically, paramedics and nurses might be perceived more as peers, as they are frequently available for consultation in the NGO clinics. In contrast, interactions with physicians typically occur in more formal settings, where they may more likely be perceived as expert service providers and their words afforded greater value.

Drawing smokers' attention to their smoking behaviour is a form of feedback to the patient and might in itself be sufficient to effect behaviour change when administered by a trusted and valued health professional. While more intensive, multifaceted counselling interventions may be the ideal, they are not always feasible in busy primary care or clinical settings, particularly in low-resource environments, where health care providers' time is at a premium. From a public health perspective, even given a small effect size for facilitation of smoking cessation through brief physician intervention, the net effect can still be substantial, provided large numbers of physicians follow such a practice systematically [59].

These results should be considered within the context of several study limitations. First, although this study surveyed a large group of participants from multiple NGO outreach networks across the Kathmandu Valley, findings are specifically representative of PLWHA falling within the network of our partner NGOs. Importantly, our sample contained only two non-heterosexual participants, though men who have sex with men represent an important risk group in the HIV epidemic nationally. Second, this study relies on self-report of all measures, leaving room for several types of bias. Because information was collected through face-to-face interviews, a social desirability bias may have been introduced, though a confidential and sensitive method of survey administration was designed to limit this tendency. Although we did not verify smoking status biochemically for those who reported being nonsmokers, there is empirical evidence that self-report is a sufficient, reliable, and valid means to assess smoking status [60].

Third, measurement of smoking status assessment experiences in this study only covered the existence of such an interaction and did not address potential variability in the specific content or quality of the discussions on smoking taking place between patients and their physicians. Finally, though previous research has established that stage of behaviour change effectively predicts both smoking cessation attempts and actual cessation [29,30], the tangible outcome variable of this study is readiness to quit rather than eventual quitting success. Further research will be needed to gain a better understanding of the different nuances to effective delivery of smoking status assessments in HIV care settings as well as the special needs HIV-positive smokers may have in actually enacting and maintaining smoking cessation.

## Conclusions

Roughly one-third of HIV-positive smokers residing in the Kathmandu Valley, Nepal, are at the contemplation or preparation stage of smoking behaviour change, with rates significantly higher among those whose physicians have asked about their smoking status during any clinical interaction over the past year. Although longitudinal studies will be necessary to draw decisive conclusions, our findings suggest an important role for physicians in raising awareness and catalyzing change among their currently smoking HIV-positive patients. Particularly in an era of pervasive ART, the prevention of chronic, tobacco-related disease represents an increasingly critical component of HIV medicine globally. Even through the most minimal investment of time and resources, HIV health care systems show promise as a gateway for effective delivery of smoking cessation interventions to the critical though neglected population of HIV-positive smokers.

## Competing interests

The authors declare that they have no competing interests.

## Authors' contributions

RMA conceived of the study, contributed to the study design, conducted the statistical analyses, and drafted the article. KCP helped to conceptualize the study and contributed to the design of the study, the interpretation of results, and the revisions of the article. RMA, KCP, and KPT oversaw the data collection. JK and BDP assisted in the implementation of the study. MJ monitored and supervised the study progress. All authors contributed to critical revision of and approved the final manuscript.

## Pre-publication history

The pre-publication history for this paper can be accessed here:

http://www.biomedcentral.com/1471-2458/11/677/prepub
